# Impact of Microplastics and Nanoplastics on Human Health

**DOI:** 10.3390/nano11020496

**Published:** 2021-02-16

**Authors:** Maxine Swee-Li Yee, Ling-Wei Hii, Chin King Looi, Wei-Meng Lim, Shew-Fung Wong, Yih-Yih Kok, Boon-Keat Tan, Chiew-Yen Wong, Chee-Onn Leong

**Affiliations:** 1 Centre of Nanotechnology and Advanced Materials, Faculty of Engineering, University of Nottingham Malaysia Campus, Jalan Broga, Semenyih 43500, Malaysia; 2 Center for Cancer and Stem Cell Research, Institute for Research, Development and Innovation (IRDI), International Medical University, Kuala Lumpur 57000, Malaysia; lingweihii@imu.edu.my (L.-W.H.); 00000028295@student.imu.edu.my (C.K.L.); weimeng_lim@imu.edu.my (W.-M.L.); 3 School of Postgraduate Studies, International Medical University, Kuala Lumpur 57000, Malaysia; 4 School of Pharmacy, International Medical University, Kuala Lumpur 57000, Malaysia; 5 Center for Environmental and Population Health, Institute for Research, Development and Innovation (IRDI), International Medical University, Kuala Lumpur 57000, Malaysia; shewfung_wong@imu.edu.my (S.-F.W.); yihyih_kok@imu.edu.my (Y.-Y.K.); boonkeat_tan@imu.edu.my (B.-K.T.); wongchiewyen@imu.edu.my (C.-Y.W.); 6 School of Medicine, International Medical University, Kuala Lumpur 57000, Malaysia; 7 School of Health Sciences, International Medical University, 126, Jalan Jalil Perkasa 19, Bukit Jalil, Kuala Lumpur 57000, Malaysia

**Keywords:** nanoplastics, nanotoxicity, nanomaterials, toxicology, plastics, health impacts, environmental impacts, pollution

## Abstract

Plastics have enormous impacts to every aspect of daily life including technology, medicine and treatments, and domestic appliances. Most of the used plastics are thrown away by consumers after a single use, which has become a huge environmental problem as they will end up in landfill, oceans and other waterways. These plastics are discarded in vast numbers each day, and the breaking down of the plastics from micro- to nano-sizes has led to worries about how toxic these plastics are to the environment and humans. While, there are several earlier studies reported the effects of micro- and nano-plastics have on the environment, there is scant research into their impact on the human body at subcellular or molecular levels. In particular, the potential of how nano-plastics move through the gut, lungs and skin epithelia in causing systemic exposure has not been examined thoroughly. This review explores thoroughly on how nanoplastics are created, how they behave/breakdown within the environment, levels of toxicity and pollution of these nanoplastics, and the possible health impacts on humans, as well as suggestions for additional research. This paper aims to inspire future studies into core elements of micro- and nano-plastics, the biological reactions caused by their specific and unusual qualities.

## 1. Introduction

Worldwide, plastic use is growing year by year, with current figures showing plastic production exceeding 368 million tons in 2019 [1]. Furthermore, the waste produced is not disposed in the correct way. As plastic pervades every aspect of life and then breaks down into smaller particles, the possible impacts of micro- and nano-plastics on the human body and the environment are of global concerns [2].

Plastics are made of natural materials that have undergone several chemical processes and physical reactions. The main processes used are polymerization and polycondensation, during which the core elements are fundamentally transformed into polymer chains [3]. This process is rarely reversible; the plastics must go through more chemical processes in order to be recycled into new types of plastics [4]. The use of industrial additives, such as pigments, plasticizers and stabilizers, allows plastics to be engineered to various application requirements [5]. Due to the chemical stability of the conventional plastics, environmental accumulation is on the rise.

Once disposed of, plastic waste is exposed to biological, chemical and environmental elements, and will break down into huge amounts of microplastics (measuring < 5 mm) and nanoplastics (<0.1 µm) [6,7,8,9]. Previous studies into plastic waste have looked at the effect of microplastics on the environment, and this has been widely discussed in both the scientific community and the media, including appeals for institutional policies to implement hazardous classifications of the harmful plastics [10]. However, there is very little research into the quantities, varieties and toxicity of nanoplastics and the impacts on human health. A solitary microplastic particle will break down into billions of nanoplastic particles suggesting that nanoplastic pollution will be prevalent across the globe [11,12,13]. It is probable that nanoplastics are more damaging than microplastics as they are small enough to permeate through biological membranes. Despite of this, the potential human health effects of nanoplastic exposure remains under-studied.

Hence, this review aims to explore thoroughly on how nanoplastics are created, how they behave/breakdown within the environment, levels of toxicity and pollution of these nanoplastics, and the possible health impacts on humans, as well as suggestions for additional research.

## 2. Sources and Fate of Microplastics and Nanoplastics in the Environment

Over 80% of microplastics are produced on land, with less than 20% originating from the sea. As microplastics are uniquely light, indestructible and able to float, they can travel far across the globe [14,15]. The majority of plastics that polluting the marine environment originate from terrestrial sources, fishing and other aquaculture activities, and from coastal tourism [14,16,17]; indeed, it is estimated that over 800 million tons of plastics in the sea originated from land [18]. As micro- and nanoplastics are incredibly small, wastewater treatment processes are not able to filter them out and, therefore, such plastic particles will be introduced into the rivers and oceans, as well as the fresh water supply system [19]. Furthermore, micro- and nanoplastics are present in soil and, through natural erosion, they will also get into rivers and oceans in this way [20]. Figures from the United Nations Environment Program show that 275 million tons of plastic waste were produced in 2010 with an estimated 4.8–12.7 million tons leaching way into the water systems [21].

**Figure 1:**
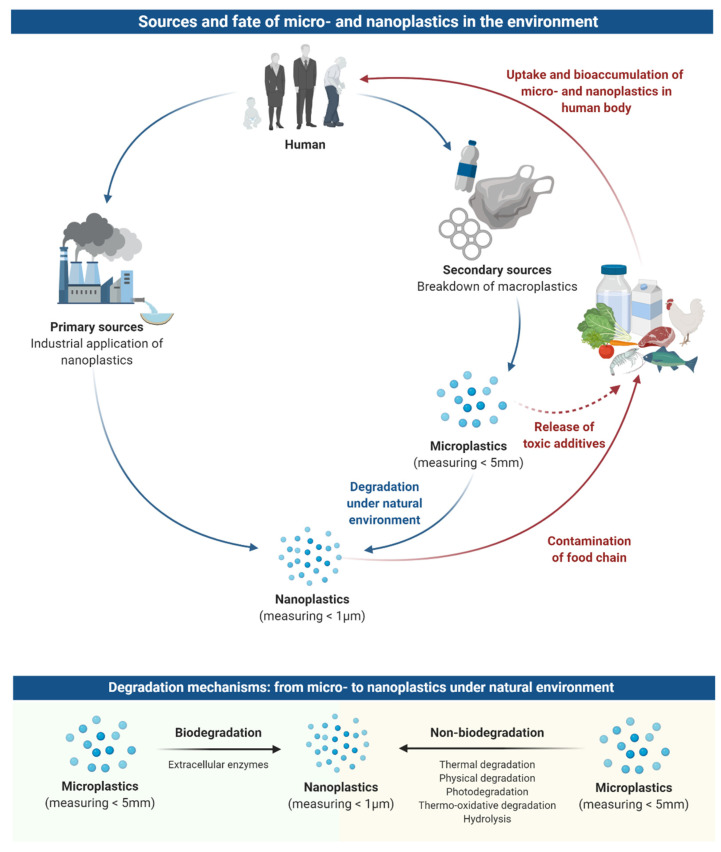
Sources and fate of micro- and nanoplastics in the environment. Micro- and nanoplastics are generated from primary and secondary sources through consumers and industries. Macroplastic products that disintegrate into micron-sized can break down into nanoplastics via biodegradation or non-biodegradation process. Both micro- and nanoplastics can occur in both aquatic and terrestrial environment, and eventually enter the food chain and water supplies, leading to the uptake and bioaccumulation of these plastic particles in the human body.

Micro- and nanoplastics are generated from both primary and secondary sources (Figure 1) [22]. Primary sources are those that deliberately created micro- and nanoplastics for consumer and industrial uses, such as exfoliants in cleansers, cosmetics, as drug delivery particles in medicines, and industrial air blasting [15]. Macroplastic products that disintegrate into micron-sized and smaller particles are the secondary source of micro- and nanoplastics; they occur both terrestrially and in the aquatic environment [15].

Plastics can break down into micro- and nanoplastics in various ways which can be defined as either via biodegradation or non-biodegradation process (Figure 1). Processes such as thermal degradation, physical degradation, photodegradation, thermo-oxidative degradation, and hydrolysis are all examples of non-biodegradation [23,24,25,26]. Thermal, or heat degradation is a non-natural, commercial process whilst physical degradation, through weathering, causes larger plastics to be fragmented into smaller pieces. On the other hand, hydrolysis and photodegradation are naturally occurring chemical processes which use water molecules, and UV-visible light, respectively, to break down the chemical bonds in plastics, converting them into monomeric forms. Plastic non-biodegradation processes decompose polymeric structures, altering their mechanical properties, and increasing their specific surface area, resulting in enhanced physical-chemical reactions and interactions with microorganisms [27].

Environmental bacteria and other microorganisms can also mediate the biodegradation process of plastics [28]. Extracellular enzymes produced by these living organisms have the ability to break down the chemical bonds within plastics [29]. Smaller plastic particles, with altered molecular structures are created in this process, eventually resulting in nano-sized plastics; a single gram of macroplastic can yield billions of nanoplastic particles with greatly increased surface area. As there is a vast quantity of plastic entering the oceans daily, it is clear that these nanoplastics must be present in enormous quantities in the marine environment.

In addition, plastic waste fragmentation is thought to occur faster on the coast than in the oceans. One of the primary methods of degradation of plastic is oxidation triggered by solar UV irradiation. This process speeds up on the coast when plastic is more directly exposed to UV radiation and higher temperatures than when it is in the oceans [30]. Further, plastic degradation is quickened in the presence of salt at these coastal areas [31]. In comparison to terrestrial ecosystems, high saline content, along with naturally occurring microorganisms in marine areas, will cause plastics to break down at a faster rate [20].

## 3. Occurrence of Microplastics and Nanoplastics in the Food Chain

As plastic waste increases, the presence of micro- and nanoplastics in the food chain creates a risk to human health [22,32,33]. Due to their wide bioavailability and ubiquity in both aquatic and terrestrial areas, it is highly probable that micro- and nanoplastics are present in many food products.

Several studies have shown that micro- and nanoplastics enter into the human food chain in various ways: Animals consuming them in their natural environment [34]; contamination during the food production processes [35]; and/or through leaching from plastic packaging of the food and drinks [36]. To date, micro- and nanoplastic fragments have been detected in honey, beer, salt, sugar, fish, shrimps and bivalves [37,38,39,40,41,42]. Experimental sampling using Fourier-transform infrared spectroscopy (FTIR) performed on tap, bottled and spring waters showed that microplastics are present in all these water sources. Tap water from 159 global sources was tested and 81% were found to contain microplastic particles measuring less than 5 mm [43]. Tests were conducted on 259 individual bottles of water from 11 different brands and 27 different batches, and the results demonstrated that 93% contained microplastic particles [36]. Statistics show the following average levels of microplastic pollution in food: seafood = 1.48 particles/g, sugar = 0.44 particles/g, honey = 0.10 particles/g, salt = 0.11 particles/g, alcohol = 32.27 particles/L, bottled water = 94.37 particles/L, tap water = 4.23 particles/L, and air = 9.80 particles/m^3^ [9,44]. From these figures, it is possible to extrapolate that the average human is consuming around 39,000 to 52,000 microplastic particles per year, with age and gender impacting the total amount. If inhalation of plastic particles is included in the figures, then the amounts rise to between 74,000 and 121,000 particles per year. Further, an individual who only ingest bottled water is potentially consuming an extra 90,000 particles in comparison to people who only drink tap water, who will ingest only 4000 extra particles [44]. These results indicate that the human food chain is, indeed, a major source of microplastic consumption by humans.

There are currently no data regarding the presence of nanoplastics in food as the analytical tools are not yet available [9,45]. It appears clear, though, that nanoplastics will occur in the food chain due to the degradation of microplastic waste [25,45]. Scientific tests on polystyrene drinking cup lids showed that nanoplastics were formed over time as the material broke down [28]. There is also evidence to suggest that microbial degradation will occur in oceans due to the presence of hydrocarbon-degrading microorganisms that have been shown to flourish on plastic waste, forming a “plastisphere” ecosystem [26]. The huge scale of plastic waste dispersal in the oceans indicates that microplastics will continue to degrade once they enter the sea, forming more nanoplastic particles [46]. There are also a number of products that use commercially manufactured nanoplastics, and these will also become plastic waste in the seas and on land, and eventually find their way into the food supply chain [9,45].

## 4. Uptake and Bioaccumulation of Microplastics and Nanoplastics in the Human Body

**Figure 2:**
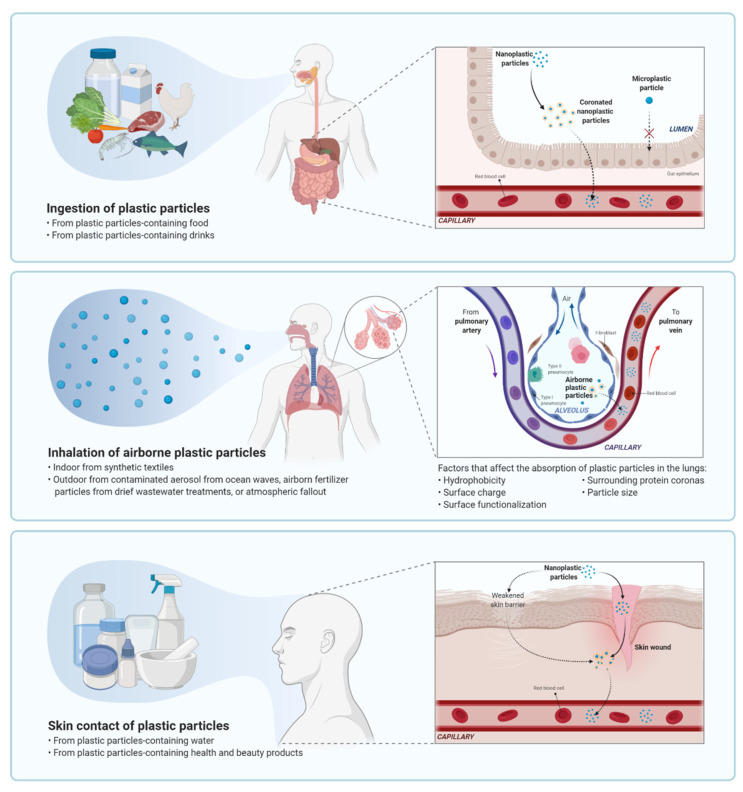
Routes of plastic particles entry into human body. There are three key routes for micro- and nanoplastics entry into the human body: Inhalation, ingestion and skin contact. Nanoplastics may interact with proteins, lipids, carbohydrates, nucleic acids, ions, and water in human body, leading to the formation of coronated nanoplastic particles for absorption. The plastic particles can enter human body through ingestion of contaminated food and water supplies, or inhalation of the airborne plastic particles that originate from synthetic textiles and polluted outdoor air. While, the skin membrane is too fine for these plastic particles to pass through, nanoplastics may penetrate through wound and weakened skin barrier, directly or indirectly.

There are three key routes for microplastics and nanoplastics to end up in the human body: Inhalation, ingestion and skin contact (Figure 2) [47,48]. Inhaled airborne microplastics originate from urban dust, and include synthetic textiles and rubber tyres [49]. As discussed above, microplastics will be ingested as they are prevalent in the food chain and water supplies [50]. While, the skin membrane was too fine for microplastics or nanoplastics to pass through, it is possible for them to enter through wounds, sweat glands or hair follicles [51]. Although all three routes contribute to the total amount of microplastics and nanoplastics present in the human body, it is the particles in seafood and the environment that constitute the greatest risk of absolute exposure. This is due to long-term weathering of polymers, leaching of polymer chemical additives, residual monomers, exposure to pollutants and pathogenic microorganisms all being active in these environments [52,53,54,55,56].

### 4.1. Gastric Exposure

Recent studies into microplastics and nanoplastics exposure and toxicity have indicated that the most significant way humans consume plastic particles is via ingestion [57]. While, there are no studies looking specifically at nanoplastic toxicity in humans, there is research showing that microplastics are being ingested through food and drink [36]. The initial analysis of human stool samples showed that plastic particles were being excreted, which supports the theory that humans are ingesting these particles via food and water. These results, along with research into ingestion uptake in environmental models, clearly show that humans will be regularly consuming microplastics and nanoplastics [58]. However, no studies have yet investigated what happens to the micro- and nanoplastic particles once they enter the gastrointestinal (GI) tract. It would be pertinent to examine their route through the GI tract and whether particles remain in the gut lumen, or they translocate across the gut epithelia.

It is unlikely that microplastics are able to permeate at a paracellular level as the relevant pores at the tight junction channels have a maximum functional size of approximately 1.5 nm [59]. It is more likely that they enter through lymphatic tissue, and it is particularly possible that they enter via phagocytosis or endocytosis and infiltrate the microfold (M) cells in the Peyer’s patches [45]. Following intraperitoneal injections in mice, the peritoneal macrophages were seen to phagocytose 1, 5 and 12 μm polymethacrylate and polystyrene particles [60,61]. Nevertheless, the results indicate that absorption via intestinal tracts in rodent models is low at 0.04–0.3% [61].

The potential of nanoplastics to permeate the gut epithelium, leading to systemic exposure in humans, is a significant issue. Historically, studies have used polystyrene nanoparticles for in vivo and in vitro tests on a variety of animals. The probable oral bioavailability level of 50 nm polystyrene nanoparticles is ten to one hundred times greater than the level of microplastics (2–7%) [62,63]. Similar to the results seen with microplastics, there is no straightforward correlation between the absorption, size and structure of nanoplastics [62]. Previous research has shown that the absorption rates of nanoparticles (50–500 nm) vary greatly across different in vitro intestinal models, with figures of 1.5–10% according to the size and chemical structure of the nanoparticles as well as the type of in vitro model used [62,64,65].

The lumen of the GI tract presents a challenge when researching nanoplastic absorption rates. Once consumed, nanoparticles undergo transformation, and this will impact absorption ability and rates. There are several molecules within the GI tract that nanoparticles may interact with, such as proteins, lipids, carbohydrates, nucleic acids, ions, and water [9]. This then leads to the nanoparticles being encompassed by a collection of proteins known as a ‘corona’ [66]. Polystyrene nanoparticles may develop into varying forms of complex coronas, according to the conditions they are in [67]. Studies have shown that protein corona changes within an in vitro model representing human digestion, and this leads to greater translocation of nanoparticles [62]. Furthermore, organic matters found in bodies of water will adhere to the surface of nanoparticles; a recent review has examined how dispersed organic materials react with metal (oxide) nanoparticles and determined that these interactions have a significant impact on agglomeration and deposition [68].

It is worth noting that most of the reported studies were based on experiments using polystyrene nanoparticle models, and excluded samples gathered from marine and terrestrial environments. Other plastics such as polypropylene (PP), polyethylene (PE), and polyethylene terephthalate (PET) are, however, the main polymeric materials present in these environments. Thus, it is critical to qualify any extrapolations made from the findings of the research discussed above, which relies solely on polystyrene. Instead, new model studies should include experiments with PP, PE and PET.

### 4.2. Pulmonary Exposure

The second most likely method of human exposure to nanoplastics is through inhalation. Indoor environments contain airborne plastic particles, primarily from synthetic textiles, leading to unintended inhalation or occupational exposure [69]. In outdoor environments, exposure could happen through breathing in contaminated aerosols from ocean waves or airborne fertilizer particles from dried wastewater treatments [57]. The alveolar surface area of the lungs is vast, being approximately 150 m^2^ and has an incredibly fine tissue barrier measuring less than 1 μm. This barrier is thin enough for nanoparticles to permeate through it and into the capillary blood system, thus, meaning that nanoparticles can disperse through the entire human body [57].

There are several negative health concerns resulting from the absorption of plastic particles, particularly micro- and nanoplastics, such as particle toxicity, chemical toxicity, and the introduction of pathogens and parasite vectors [70,71]. Particles within this range of sizes can potentially be embedded deep into the lung and then stay on the alveolar surface or translocate to other parts of the body [69,72]. The absorption of plastic particles through inhalation could lead to lung damage. There are a number of factors that affect absorption and expel micro- and nanoplastics in the lungs, such as hydrophobicity, surface charge, surface functionalization, surrounding protein coronas, and particle size [73]. In addition, research into absorption rates in animals indicate a positive correlation between occupational exposures and higher rates of pulmonary inflammation and cancer [49]. Research looking at absorption rates of polystyrene particles in alveolar epithelial cells, in vitro, suggests that absorption varies according to the size of the plastic particles [74,75,76,77].

Recent studies into the human inhalation of plastic particles have indicated that atmospheric fallout in urban areas is a significant cause of the particles [78]. The major constituent atmospheric fallout of microplastics from both urban and suburban areas of Paris was found to be synthetic fibre particles, where 29% of those fibres contained petrochemicals. By considering the average atmospheric flux of total fibres, the fibre dimensions and fibre densities, an estimated 3–10 tons of microplastics are deposited annually as a result from atmospheric fallout. Urban areas recorded double the average atmospheric flux, compared with suburban areas, with rainfall having a demonstrable impact on the observed depositions [78]. Dris et al. also examined the levels of microplastic particles in indoor and outdoor air at two private apartments and one office. The results showed a concentration of between 1 and 60 fibres/m^3^ in the indoor samples. These readings were considerably greater than the outdoor samples which had levels of between 0.3 and 1.5 fibres/m^3^. Approximately one-third of the indoor samples were of petrochemical origins, with the majority composed of polypropylene, while the rest were cellulose [79]. To date, there is no information regarding the amount or concentration of airborne nanoplastics.

### 4.3. Dermal Exposure

Health and beauty products are another key source of nanoplastics, particularly in the body and facial scrubs that are used topically on the skin [11]. Nanocarriers for drug delivery via dermal application is another important exposure route. Although there are no conclusive data showing the effects of nanocarriers, small particle size and stressed skin conditions are critical factors to skin penetration [51]. There is no current research that has specifically looked at the ability of nanoplastics to penetrate the surface of the skin. Only one study reported the likelihood of engineered nanoparticles from textiles in permeating the skin barrier at very minute quantity [80].

The skin is protected by the stratum corneum, the outermost layer, which forms a barrier against injuries, chemicals and microbial agents. The stratum corneum consists of corneocytes that are surrounded by lamellae of hydrophilic lipids including ceramide, long-chain free fatty acids and cholesterol [81]. Plastic particles may be introduced on the skin through health and beauty products, or through contact with nanoplastic-contaminated water. As micro- and nanoplastics are hydrophobic, it is predicted that absorption through the stratum corneum through contaminated water is unlikely, though plastic particles could enter the body via sweat glands, skin wounds or hair follicles [51].

Alvarez-Roman et al. looked at how plastic particles enter the body and how they are then distributed throughout the skin tissue. They used fluorescent polystyrene particles between 20 and 200 nm in diameter and skin tissue from a pig to conduct their experiment [82]. A confocal laser scanning micrograph of the skin revealed that a greater number of 20 nm polystyrene nanoplastics concentrated in the hair follicles than those of 200 nm nanoplastics. However, neither particles were able to permeate the stratum corneum in order to embed themselves into the deeper skin tissue. Campbell et al. supported these findings and established that polystyrene particles with diameters of 20–200 nm can infiltrate only the top layers of the skin at a depth of 2–3 μm [83]. Vogt et al. were able to distinguish 40 nm-diameter fluorescent polystyrene nanoparticles in the perifollicular tissue of skin explants that had been treated with cyanoacrylate follicular stripping. This work ascertained that when particles were applied transcutaneously, they were then absorbed by the Langerhans cells [84].

The mechanical production method used to manufacture the microbeads of the health and beauty products, including facial and body scrubs, increases the likelihood of the breakdown of microbeads into even more harmful nanoplastics. Hernandez et al. investigated the amount of nanoplastics present in facial scrubs containing 200 μm polyethene microbeads. The results from scanning electron microscopy confirmed that nanoparticles were observed at sizes between 24 ± 6 nm and 52 ± 14 nm. Then, they examined the chemical composition of these nanoparticles by using X-ray photoelectron spectroscopy and Fourier transform infrared spectroscopy and established that they consisted of polyethylene [11].

Data from these previous studies can be used to determine the likelihood of nanoplastics penetrating the stratum corneum. Exposure to UV rays causes skin damage, which means that the skin barrier becomes weaker [85]. A study on the effects of UV irradiation on murine skin found an increase in the skin permeation by carboxylated quantum dots. The intercellular adhesion in the irradiated skin was compromised by the perturbed expression of tight-junction-related proteins: Zonula occludens-1, claudin-1, and occludin [86]. There are a number of compounds, e.g., short chain- and long chain-alcohols, cyclic amides, esters, fatty acids, glycols, pyrrolidones, sulphoxides, surfactants and terpenes, that are used to enhance the chemical permeation of drugs and formulations through the skin barrier [87]. Widely used ingredients in body lotions, such as urea, glycerol and α-hydroxyl acids also enhanced the ability of nanoparticles to permeate the skin barrier [88].

Kuo et al. highlighted how oleic acid, ethanol and oleic acid-ethanol enhancers impact the transdermal delivery of 10 nm zinc oxide nanoparticles. They determined that each chemical had the potential to improve the effectiveness of zinc oxide nanoparticles in penetrating the skin barrier, through the multilamellar lipid regions between corneocytes [89]. Through crystalline structure analysis of various compositions of lipid lamellae in stratum corneum samples taken from human and porcine sources, Bouwstra et al. presented a three-layer “sandwich model”, which likely prevents large nanoparticles from permeating undamaged skin [81].

To summarize, both in vitro and in vivo studies, discussed above, have established that micro- and nanoplastics can be absorbed into the human body through the skin barrier. That being said, these studies all rely on polystyrene particle models. Further studies conducted with samples collected from the environment would be helpful to fully understand the permeation qualities of micro- and nanoplastics. Such samples would include a variety of plastic particles with wide-ranging characteristics.

## 5. Cellular Uptake and Intracellular Fate of Microplastic and Nanoplastic Particles

After permeating into the stratum corneum and absorbed into the human body, microplastics and nanoplastics are then able to interact with numerous target cells. The quantity of nanoparticles that are absorbed and subsequently react with cells depends on a number of factors, such as their size, surface chemistry or charge of the biological elements they encounter, including proteins, phospholipids and carbohydrates [90]. As nanoparticles absorb proteins from the human body, they create ‘protein coronas’ around themselves [91]. This means that nanoparticles that interact with organs or skin cells will usually already be surrounded with protein corona as opposed to an exposed nanoparticle. The protein coating will lead to modified characteristics of the nanoparticle. Previous in vitro experiments have determined that protein coronas will surround polystyrene nanoparticles and that this enables the nanoparticles to translocate at greater rates [62]. These studies also showed that the protein coronas would alter their form according to their environment [67] and that there were more occurences of cell interactions and increased toxicity [92]. Finally, it was shown that protein coronas around the polystyrene nanoparticles led to them being accumulated in the gut.

**Figure 3:**
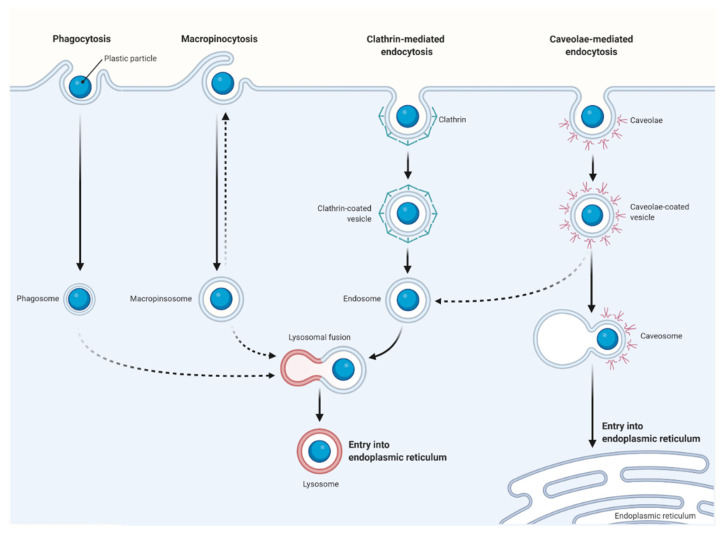
Routes of cellular uptake of plastic particles. Phagocytosis, macropinocytosis, clathrin- and caveolae-mediated endocytosis are the common endocytotic pathways that have been identified for cellular uptake of plastic particles. Micro- and nanoplastics can be absorbed by cells through different routes, of which endocytotic nanoparticle uptake is the primary route where adhesive interaction of nanoparticles (or inactive permeation of the cell membrane) with channel- or transport-protein occurs.

Microplastics and nanoplastics can be absorbed by cells via a number of routes [93]. The primary route is via endocytotic nanoparticle uptake where adhesive interaction of nanoparticles (or inactive permeation of the cell membrane) with channel- or transport-protein occurs. Several endocytotic pathways have been identified, such as phagocytosis and macropinocytosis, along with clathrin- and caveolae-mediated endocytosis (Figure 3) [94,95,96].

The initial barrier to nanoparticle incursion into the skin is the outer cell membrane. Coarse-grained molecular simulations of polystyrene (PS) particles in interacting with biological membranes revealed that polystyrene nanoparticles effortlessly penetrated the lipid bilayer membranes, causing changes to the structure of the cell membrane, ultimately disrupting the cell function [97]. Uptake inhibition studies on the absorption rate of 44 nm polystyrene particles to human colon fibroblasts and bovine oviductal epithelial cells indicated that polystyrene nanoparticles were primarily absorbed through a clathrin-independent uptake mechanism [98].

The cellular absorption of carboxylated polystyrene nanoparticles of 40 nm and 200 nm was studied using several tumor cell lines, including human cervical HeLa cells, human glial astrocytoma 1321N1, and adenocarcinomic human alveolar basal epithelial A549 in the presence of various transport inhibitors [99]. The results indicated that the nanoparticles were always absorbed by cells through an active and energy-dependent method, suggesting that the nanoparticle uptake for different cell types utilized different pathways. Actin depolymerization significantly influenced the nanoparticle uptake in HeLa and 1321N1 cell lines, while clathrin-mediated endocytosis of the nanoparticles in 1321N1 was significantly reduced by the inhibitor chlorpromazine. Nanoparticle uptake in A549 cell line via caveolae-mediated endocytosis was notably diminished due to disruption of microtubule formation by the inhibitor genistein [99].

The routes of nanoparticle absorption in human cells are dependent on the size and surface chemistry of the particles, but also vary according to the type of cell penetrated. This is seen when 120 nm polystyrene nanoparticles altered with amidine groups were seen to permeate rat alveolar epithelial monolayers using non-endocytic pathways, whilst MDCK-II cells use energy-dependent processes to absorb nanoparticles [76,100]. Macrophages and epithelial cells were found to use various combinations of endocytotic uptake mechanisms to absorb 40 nm carboxylated polystyrene nanoparticles.

Using several endocytotic pathway inhibitors, J774A.1 macrophages were shown to absorb nanoplastics via macropinocytosis, phagocytosis, and clathrin-mediated endocytosis pathways, whilst absorption in A549 cells relied on caveolae- and clathrin-mediated endocytosis pathways [101]. If nanoplastic particles are entering the human body via non-vesicular pathways, then they may be able to interact with intracellular molecules or discharge persistent organic pollutants (POPs) straight into the cytoplasm. This, in turn, implies that POPs may be stored in human cells which could have a negative toxicological impact [102].

Plastic particles enter cells via the intracellular endocytotic pathway engaging with early and late endosomes before combining with lysosomes. Polystyrene nanoparticles have been reported to accumulate in the lysosome [103], including the observance of intracellular localization of 40–50 nm polystyrene nanoparticles in A549 cells [77]. No lysosomal leakage or any fragmentation of the nanoparticles were reported when acidic conditions were applied [104].

A comparison of two types of nanoparticles made of polystyrene and mesoporous silica demonstrated clear differences in cellular uptake mechanisms in ovarian cancer cells. Data gathered demonstrated that ovarian cancer cells absorbed both types of nanoparticles with different endocytotic pathways [105]. Caveola-mediated endocytosis was the pathway used by mesoporous silica particles to permeate the cells and these then, according to the size of the particle, either remained in the lysosome (50 nm) or translocated into the cytoplasm (10 nm). On the other hand, polystyrene nanoparticles were absorbed through a caveola-independent pathway. Localized amine-modified 50 nm polystyrene particles showed toxicity to the lysosome after 4–8 h, whereas 30 nm carboxyl-modified polystyrene particles showed no signs of toxicity and did not enter cells via the standard acidic endocytotic route.

Previous research has also determined that cytotoxicity is greater with a positive surface charge and this also leads to increased absorption of nanoparticles via non-specific binding, where they end up on the negatively charged sugar moieties on cell surfaces. In contrast, the repellent interactions of negatively charged particles will impede endocytosis [103,106]. Studies have determined that there are several cellular absorption pathways and intracellular localization of polystyrene nanoparticles depending on their physicochemical characteristics. Despite this, there is little quantitative data concerning how nanoplastics enter cells and their eventual fates.

The size of plastic particles also affects their interaction with human cells [107]. Due to their high specific surface areas, the nanoparticle-cell interaction is vastly different compared to larger particles. Furthermore, the charge of the particle can also affect its interaction with the cell and its structure [107]. Since most of the in vitro studies described here have used polystyrene particles, it is difficult to extrapolate the results to other kinds of plastic particles. It is important that future research looks at cellular uptake and behaviours of other types of plastic particles.

## 6. Potential Toxic Effects of Microplastics and Nanoplastics on Human Health

Several in vitro and in vivo studies have shown that micro- and nanoplastics were able to cause serious impacts on the human body, including physical stress and damage, apoptosis, necrosis, inflammation, oxidative stress and immune responses (Table 1) [108,109,110,111].

### 6.1. Inflammation

An in vitro study using various sizes of polystyrene particles found that larger particles (202 nm and 535 nm) produced inflammatory effects on human A549 lung cells. There was higher IL-8 expression by the lung cells treated with the larger particles, in comparison with the same cells exposed to 64 nm particles [112]. Furthermore, unaltered or carboxylated polystyrene nanoparticles brought on a substantial up-regulation of IL-6 and IL-8 genes in human gastric adenocarcinoma, leukemia, and histiocytic lymphoma cells, which suggests that the increase in inflammatory reactions to polystyrene particles is likely to be due to the constitution of the particle, or down to simple particle occurrence rather than because of the particle charge [113,114].

A study on the impact of carboxylated and amino-modified polystyrene particles (120 nm) on the polarization of human macrophages into M1 or M2 phenotypes revealed no change in the expression of M1 markers like CD86, NOS2, TNFα, and IL-1β [115]. However, the introduction of both types of nanoparticles negatively impacted the expression of scavenger receptors CD163 and CD200R, and the release of IL-10 by M2 cells. There was a reduction of *Escherichia coli* phagocytosis by both M1 and M2 macrophages with the introduction of amino-modified particles. On the other hand, phagocytosis by M2 was unaffected by the carboxylated particles. The carboxylated particles also caused increases in the protein mass in M1 and M2, enhanced the release of TGFβ1 by M1, and heightened levels of ATP in M2 [115]. Similarly, in vitro study also shown that unmodified polyethylene particles measuring between 0.3 and 10 μm caused murine macrophages to produce significant levels of cytokines, such as IL-6, IL-1β, and TNFα [116,117].

Furthermore, a number of previous studies indicated that when polyethylene components are used as prostheses, they can fragmentize as a result of wear and tear, and form debris in the joints [118,119,120]. The polyethylene wear particles trigger the TNFα and IL-1 pro-inflammatory factors, as well as pro-osteoclastic factors, including the receptor activator of NF-κB ligand (RANKL), which causes periprosthetic bone resorption and could eventually result in the patient losing the prosthesis [121]. High levels of plastic particles measuring between 0.2 and 10 μm have been observed in the periprosthetic tissue of ultrahigh molecular weight polyethylene-based implants. In addition, the presence of macrophages in the tissues in the vicinity of the implant area indicates the stimulation of the inflammatory response [121,122,123]. A study of failed titanium alloy total hip arthroplasty cases found that a majority of wear debris in the interfacial membranes consisted of polyethylene particles with an average diameter of 530 nm [124]. To overcome the observed negative effects, surgeons are now increasingly employing metal-on-metal joint replacements.

### 6.2. Oxidative Stress and Apoptosis

A number of in vitro studies have shown that different polystyrene nanoparticles can induce oxidative stress, apoptosis and autophagic cell death in cell context-dependent manner. For instance, amine-modified polystyrene nanoparticles were shown to interact and aggregate with mucin strongly, and induce apoptosis of mucin- and non-mucin-secreting intestinal epithelial cells [126]. Cationic polystyrene nanoparticles were shown to induce reactive oxygen species (ROS) production and endoplasmic reticulum (ER) stress in mouse macrophages and lung epithelial cells via aggregation of misfolded protein, leading to autophagic cell death of RAW 264.7 mouse macrophages and BEAS-2B lung epithelial cells [127,128]. While, unmodified or functionalized polystyrene was shown to induce apoptosis in several human cell types, including primary human alveolar macrophages (MAC), primary human alveolar type 2 (AT2) epithelial cells, human monocytic leukemia cell line (THP-1), human immortalized alveolar epithelial type 1 cells (TT1), human colon carcinoma cells (Caco-2), and human lung cancer cells (Calu-3) [129,130,131,132]; and polystyrene nanoparticles were shown to regulate ROS via long non-coding RNAs (e.g., *linc-61, linc-50, linc-9, and linc-2*) in *Caenorhabditis elegans* [142].

Despite the toxic effects observed in the in vitro models, no obvious severe toxicity was observed in liver, duodenum, ileum, jejunum, large intestine, testes, lungs, heart, spleen, and kidneys of mice following oral exposure of a mixture of microplastics [139]. While, other studies have demonstrated that oral exposure (either through oral gavage or drinking water) caused liver inflammation [125], neurotoxic responses [125], reduced body and liver weight [140], reduced mucin excretion in colon [134,140], altered amino acid and bile acid metabolism [134,135], and altered microbiota composition [134,140,141,143]. Interestingly, some of the effects such as altered lipid metabolism was observed even in the offspring of the mice following microplastic exposure [141].

### 6.3. Metabolic Homeostasis

In addition to the induction of inflammation and apoptosis, recent studies have revealed that microplastics and nanoplastics can impair cellular metabolism in both in vitro and in vivo models. Polystyrene-based nanoparticles influence signaling systems in airway epithelial cells due to nanoparticle-cytoplasmic membrane interactions. After exposure to negatively charged carboxylated polystyrene nanoparticles measuring 20 nm, basolateral K^+^ ion channels were found to be activated in human lung cells [136]. The nanoplastic particles caused persistent and concentration-dependent increases in short-circuit currents by the activation of the ion channels and the stimulation of Cl^−^ and HCO_3_^−^ ion efflux [136].

Furthermore, 30 nm polystyrene nanoparticles induced large vesicle-like structures in the endocytic route in macrophages and human cancer cell lines A549, HepG-2, and HCT116. As a result, vesicle transport and the distribution of proteins involved in cytokinesis are blocked, thus stimulating the formation of binucleated cells [137]. In addition, acute oral exposure to positively charged polystyrene nanoparticles has the potential to disrupt intestinal iron transport and cellular uptake [138].

When mice were fed with pristine polystyrene microparticles (5 µm and 20 µm) for 28 days, the microplastics were found to be distributed in the liver, kidneys and gut, with larger particles dispersed regularly across all tissues, while the smaller particles found at higher concentration in the gut [125]. Inflammation and lipid droplets were also evident in the histopathological analysis. There was evidence that showed microplastic accumulation in murine tissue caused impairment of energy metabolism, lipid metabolism, oxidative stress and neurotoxic responses. There were decreases noted in hepatic levels of ATP, total cholesterol and triglycerides, as well as reduction in catalase activity, whereas increases were observed in the activity of several biomarkers (LDH, SOD, GSH-Px and AchE) [125,139,140].

Furthermore, pregnant mice exposed to microplastics via ingestion developed gut microbiota dysbiosis, intestinal barrier dysfunction and metabolic disorders. The effects of microplastics exposure at the maternal level also conferred permanent altered metabolism in the F1 and F2 generations [135,141]. The key results from these studies showed: (1) Change in the gut microbiota; (2) change in the intestinal barrier where less mucus was secreted and lower levels of ion transporter gene expression; and (3) alterations to lipid/fatty acid metabolism, as demonstrated by the differences in serum and liver triglyceride and total cholesterol levels [134,135,140,141].

## 7. Leaching of Toxic Chemicals from Plastics

As discussed above, plastics usually contain chemicals from the raw monomers and various types of additives to improve their properties. In addition, plastics also absorb chemicals from their surroundings [144,145]. As a result, these chemicals have the potential to leach from the polymer and into the environment around them. For example, polycyclic aromatic hydrocarbons (PAHs) have been shown to be adsorbed by microplastics and causing various toxic effects when ingested by various organisms [145]. Chemical species diffuses from the interior of a particle to its surface, leaching into the surrounding environment, and is possibly driven by a gradient function. Although these chemical species are transient and degrade rapidly in the human body, these plastic particles provide a durable ‘reservoir’ for chemical leaching into tissues and body fluid [146].

**Figure 4:**
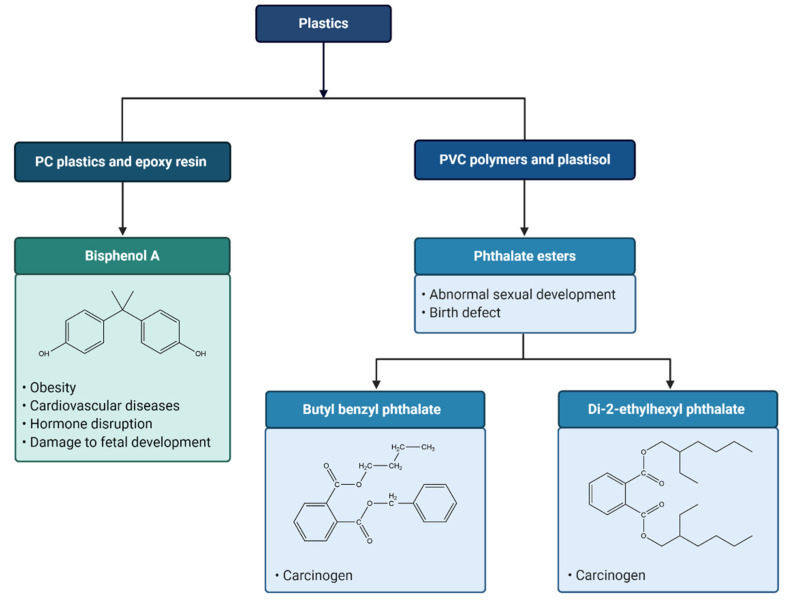
Overview of the toxic effects of chemicals leaching from plastics. Plastics are made up of different chemical compositions, in which some are hazardous that can leach to the surroundings upon degradation. Plastics typically contain additives that can improve their properties, such as durability and elasticity. The leaching of these additives from plastics to the surrounding environment, not only causing harmful impacts to the aquatic environment, but also human health. For instance, bisphenol A (BPA), an industrial chemical that is widely used to make polycarbonate (PC) plastics and epoxy resin as lining layer of food and beverage containers. Studies reported that the leaching of BPA from food containers into the food and drinks can cause a series of diseases, including obesity and cardiovascular diseases. BPA also acts as a hormonal disruptor, imitating or blocking the production, action, and function of hormones in the human body. BPA also known to affect brain development in the womb, causing damage to the developing fetus. Polyvinyl chloride (PVC) polymers and plastisol generally contain phthalate esters as plasticizers, in order to increase their durability and flexibility. Human exposure to phthalate esters has been shown to associate with abnormal sexual development and changes in the levels of sex hormones. Additionally, studies have demonstrated that some phthalate esters such as butyl benzyl phthalate (BBP) and di-2-ethylhexyl phthalate (DEHP) can increase tumor incidence in human, representing potential carcinogens.

To date, toxic chemical additives in plastic that are known to affect human health include bisphenol A (BPA), phthalates, triclosan, bisphenone, organotins and brominated flame retardants (BFR) (Figure 4) [147]. Although limited information is available whether these additives leach into the biological tissues directly, certain additives, such as nonylphenol and BPA, are found to be ingested by marine biota [148]. In particular, exposure to leached BPA, an additive that is commonly used to make polycarbonate (PC) plastics and epoxy resin as lining layer of food and beverage cans, has been shown to cause endocrine disorders and impact human health [147,149,150].

Importantly, studies have found that BPA will leach from PC into food and drinks [147,151,152], and that the toxicity of BPA causes changes in liver function and insulin resistance, damage of a developing fetus and modification of the reproductive system and neurological functions [153]. BPA acts as an agonist for estrogen receptors and inhibits thyroid hormone-mediated transcription by acting as an antagonist [154], and alters pancreatic beta cell function [155]. Increased likelihood of developing obesity and cardiovascular diseases [156,157,158], and several other reproductive and developmental issues have been noted when humans are exposed to BPA at concentrations of 0.2–20 ng/mL [147].

Phthalate esters are used as plasticizers in the manufacturing of PVC polymers and plastisol to achieve enhanced flexibility and durability [159]. Human exposure to phthalate esters are potentially harmful and may cause abnormal sexual development and birth defects [160]. Additionally, butyl benzyl phthalate (BBP) has been named as a probable carcinogen, and di-2-ethylhexyl phthalate (DEHP) has been cited as a possible carcinogen by U.S. EPA [15].

## 8. Conclusions

While, microplastics and nanoplastics are widely studied in the context of the marine environment, we have only recently recognized the potential human exposure pathways. Following exposure, uptake is plausible via ingestion and/or inhalation. The toxicity assessments of micro- and nanoplastics on human are mainly focusing on gastrointestinal and pulmonary toxicity, which involve oxidative stress, inflammatory reactions, and metabolism disorders.

Based on the findings of recent studies, further research is needed to investigate the potential mechanisms of micro- and nanoplastics toxicity in human. Moreover, it is important to understand whether microplastics and nanoplastics can be further degraded after ingestion under the acidic conditions in the gut or inside the lysosomes of the cells. Hence, the long-term fate of the ingested microplastics and nanoplastics in human body warrant further investigation.

Unfortunately, the accurate assessment of human exposure to nanoplastics remains a scientific challenge due to the lack of validated methods, certified reference materials, and standardization across the analytical procedures used [161,162]. Notably, most of the reported studies were conducted using polystyrene due to its ease in synthesis and processing into nanoparticles, while the most common commercial used of plastics are polyolefins (e.g., polyethylene and polypropylene), polyesters, and polyurethanes. Given the large variety in particle size, shape and chemical composition of plastics, the potentially hazardous effects of different types of micro- and nanoplastics to human health remain largely unknown [163]. Therefore, we recommend that future research should focus on the understanding of the potential hazards and risks of chronic exposure to diverse micro- and nanoplastics at relevant concentrations.

## Figures and Tables

**Table 1:** Summary of potential toxic effects of micro- and nanoplastics on human health.

Toxic Effects	Characteristics of Plastic Particles	Particle Size	Details	References
**Inflammation**	Polystyrene particles	202 nm and 535 nm	Upregulation of IL-8 expression. Induced inflammation in human A549 lung cells.	[112]
	Unaltered/Carboxylated polystyrene nanoparticles	20 nm, 44 nm, 500 nm, and 1000 nm	Upregulation of IL-6 and IL-8 expression. Enhanced inflammation in multiple human malignancies.	[113,114]
	Carboxylated and amino-modified polystyrene particles	120 nm	Altered expression of scavenger receptors. M2 cells increased IL-10 production. Increased TGFβ1 (M1) and energy metabolism (M2).	[115]
	Unaltered polyethylene particles	0.3 μm, 10 μm	Increased the secretion of IL-6, IL-1β, and TNFα in murine macrophages.	[117]
	Polyethylene particles from plastic prosthetic implants	0.2 μm and 10 μm	Induced the expression of TNFα, IL-1, and RANKL. Resulted in periprosthetic bone resorption.	[121]
			Induced inflammatory response at the implant area.	[121,122,123]
	Polystyrene microplastics particles	5 μm and 20 μm	Induced inflammation in the liver. Induced adverse effects on neurotransmission.	[125]
**Oxidative stress and apoptosis**	Amine-modified polystyrene nanoparticles	60 nm	Strong interaction and aggregation with mucin. Induced apoptosis in all intestinal epithelial cells.	[126]
	Cationic polystyrene nanoparticles	60 nm	Induced ROS generation and ER stress Induced autophagic cell death of mouse macrophages and lung epithelial cells.	[127,128]
	Unaltered or functionalized polystyrene	20 nm, 40 nm, 50 nm, and 100 nm	Induced apoptosis of several human cell types.	[129,130,131,132]
	polyvinyl chloride (PVC) and poly (methyl methacrylate) (PMMA)	120 nm, 140 nm	Reduced cell viability with a reduction of ATP and increase of ROS concentrations.	[133]
**Metabolic homeostasis**	Pristine and fluorescent polystyrene microplastics	5 µm	Changes in amino acid and bile acid metabolism. Induced gut microbiota dysbiosis and intestinal barrier dysfunction.	[134,135]
	Anionic carboxylated polystyrene nanoparticles	20 nm	Altered ion channel function and ionic homeostasis Activated basolateral K^+^ channels. Induced Cl^−^ and HCO^3−^ ion efflux.	[136]
	Polystyrene nanoparticles	30 nm	Blocked vesicle transport and the distribution of cytokinesis-associated proteins.	[137]
	Cationic polystyrene nanoparticles	50 nm and 200 nm	Disrupted intestinal iron transport and cellular uptake.	[138]
	Pristine polystyrene microparticles	5 µm and 20 µm	Reduction in hepatic ATP levels. Impairment of energy metabolism.	[125,139,140]
	Microplastics	0.5 µm and 5 µm	Metabolic disorder associated with gut microbiota dysbiosis and gut barrier dysfunction. Increased the risks of metabolic disorder in the offspring.	[135,141]
